# Sense of Belonging as an Important Factor in the Pursuit of Physics: Does It Also Matter for Female Participants of the German Physics Olympiad?

**DOI:** 10.3389/fpsyg.2020.548781

**Published:** 2020-10-22

**Authors:** Antonia Ladewig, Melanie Keller, Uta Klusmann

**Affiliations:** ^1^ Department of Physics Education, IPN – Leibniz Institute for Science and Mathematics Education, Kiel, Germany; ^2^ Department of Educational Research and Educational Psychology, IPN – Leibniz Institute for Science and Mathematics Education, Kiel, Germany

**Keywords:** sense of belonging, stereotype threat theory, gender, expectancy-value theory, science competitions

## Abstract

This paper focuses on stereotype threat and its effects on sense of belonging in the German Physics Olympiad science competition. Participants completed questionnaires about sense of belonging, stereotype endorsement, interest, and self-concept in physics, as well as about value and success expectations of studying physics in college. Female participants who endorsed negative stereotypes about female talent for physics felt less sense of belonging to physics. This effect did not manifest for male participants. Sense of belonging to physics significantly predicted value and success expectations for studying physics in college beyond what is predicted by interest and self-concept in physics. These findings suggest that sense of belonging is influenced by stereotype threat, which was shown to cause gender differences in science. Nevertheless, sense of belonging could be included into the expectancy-value theory based on its predictive impact on value and success expectations of studying physics.

## Introduction

Lower interest in science, technology, engineering, or mathematics (STEM) careers and often lower numbers of girls in science precipitated much research on gender differences in science, applying different rationales and theoretical approaches (e.g., Aronson et al., 1999; Schmader et al., 2004; Flore and Wicherts, 2015; Kang et al., 2019). Declining interest in science over the course of secondary school seems widely acknowledged (see, for instance, Potvin and Hasni, 2014a; Potvin et al., 2018) with only few contradicting findings (see, for instance, Kang et al., 2019). However, a study by Sadler et al. (2012) paints a more nuanced image by taking gender into account: in contrast to boys’ stable interest in STEM careers, girls’ interest declines during high school. Aside from a lower interest, women and girls also face various stereotypes regarding the environment, demands to succeed, and female talent in science (see Cheryan et al., 2015). Consequently, women less often choose most science domains in school, college, and careers (Kahn and Ginther, 2015; Miller and Wai, 2015; Su and Rounds, 2015; Wang et al., 2015; Cheryan et al., 2017). This predominance of men is especially apparent in physics. As an example, in recent years, only about 30% of the participants in the extracurricular science competition German Physics Olympiad have been female and consistently continue to drop out of the competition earlier than their male opponents. This underrepresentation of females has led to speculations whether the science Olympiads in fact offer equitable participation for males and females or simply perpetuate the gender gap (see Steegh et al., 2019). In any case, as the Physics Olympiad shows the typical male dominance in its participation numbers, it is an environment that somewhat represents the normal physics world – talented and interested girls want to pursue physics but then are deterred from that path, unable or unwilling to perform at their full potential.

Of the various approaches that try to explain the reasons for the gender gap, two figures very prominently. First is stereotype threat theory. Stereotype threat theory explains the behavior of stereotyped minorities including females in sciences based on negative stereotypes that affect behavior and performance of the minority group due to their social identification with the stereotyped group (e.g., Spencer et al., 1999; Steele et al., 2002). The impact of stereotype threat has not yet been analyzed regarding voluntary participation in extracurricular activities. Female participants of the German Physics Olympiad are a very selective sample. They have taken a first step toward a physics career by freely choosing to participate in a physics setting. Researching if they are nevertheless suffering from stereotype threat and, consequently, its negative effects is thus of interest. Second, an approach from motivational theories could help in explaining the gender gap. Among others, the expectancy-value theory (e.g., Eccles et al., 1983; Eccles, 2009) explains career decisions through achievement-related choices. By connecting stereotype threat and the expectancy-value theory, an explanation is sought of how stereotype threat might interfere in the process of forming a career choice – even when females are interested in physics and engaging in physics activities.

This study focuses on a sense of belonging as a central factor in girls’ physics motivation by connecting the theories of expectancy-value and stereotype threat in one model. The addressed research questions are:

1. Are girls in a physics environment adversely affected by stereotype threat’s consequences, in this case analyzed as having lower sense of belonging due to prevalent science gender stereotypes?
2. Does sense of belonging influence success expectations and value of studying physics?

We chose to research these questions using the German Physics Olympiad as a prototypical physics environment.

## Theoretical Background

### Sense of Belonging in Educational Settings

Humans are motivated by the need to form social attachments and feel belonging to others (Baumeister and Leary, 1995). This need is described by sense of belonging, which in educational settings typically includes feelings about school, experiences and relationships with fellow students and teachers (Allen et al., 2018). Good et al. (2012) defined it as the feeling of membership in a group and acceptance and valuation by its members. In school, sense of belonging at school is the relevant belonging. Sense of belonging at school describes the feeling of belonging specifically within the school environment and was found to be influenced by a wide range of factors, such as academic achievements (see Allen and Bowles, 2012), self-efficacy, and self-concept (Chiu et al., 2016). Additionally, it also positively influences motivation and achievement (Goodenow, 1993). Similar results were shown for belonging in college as well as belonging’s close association with task value (Freeman et al., 2007). Students with higher belonging to their college achieved higher results, felt more academically competent, and also experienced more positive self-worth (Pittman and Richmond, 2007). Results of Freeman et al. (2007) indicate that the main contributor to college belonging is the perception of how accepted one is socially by other students and college staff.

This importance of social connections can also be seen when analyzing the structure of sense of belonging. Belonging in school can be divided into academic belonging and social belonging (Green et al., 2016), whereby academic belonging is feeling belonging or acceptance within a field. Social belonging, on the other hand, is feeling membership to the group one is participating in in a certain environment. For belonging in college, five central factors were found: perceived support by peers, perceived support or comfort by faculty, perceived comfort in the classroom, perceived isolation, and empathetic faculty understanding (Hoffman et al., 2002). Taking this into account, academic belonging should be a relevant factor for school, college, and extracurricular science activities.

Intentions to continue in a field, choosing it in college or as a career and belonging are also closely connected. When belonging is felt not just within school in general, but to a certain domain, it influences students’ intention to remain in a domain through college (e.g., Good et al., 2012). It is problematic that belonging was found to decrease during middle school (Anderman, 2003), seeing as identity in a domain is relevant for career choices (Hazari et al., 2010) and mainly formed in school. Thereby, the formation of an identity interacts with belonging in the school context. Low belonging at school thus might negatively impact the formation of a strong identity as well as career choices in science. This is also the case in college, where feeling that one belongs there based on one’s own ability in the field predicts women’s intention of remaining in the field (Banchefsky et al., 2019). As the associated ability beliefs in a field are correlated with belonging (Deiglmayr et al., 2019), researching belonging in different social environments is especially important to understand the formation of career choices. Deiglmayr et al. (2019) showed that females in mathematics and physics had higher beliefs that promote brilliance or talent as the determinant of success – domains in which women also report more uncertainty of belonging. Students’ ability beliefs are also associated with the actual gender ratio in a field, meaning that fewer women in a field and higher beliefs about need of brilliance to succeed are connected (Bailey et al., 2019). Females in science thus appear to be at a disadvantage for forming belonging that could positively influence their career decisions.

A possible explanation of the connection of belonging and career choices can be found in school, where belonging also predicts value and success expectations for middle school subjects (Goodenow, 1993) and higher belonging goes along with higher perceived utility and intrinsic values of school when experienced in high school (Gillen-O’Neel and Fuligni, 2013). The expectancy-value model of Eccles et al. (1983) (e.g., Eccles, 2009) includes value and success expectations as two central elements. Based on the assumption that success expectations and the value assigned to a task inform the choices one is making in achievement-related situations, the model includes several variables influencing these two beliefs, including gender and stereotypes (Eccles et al., 1984). Thereby, a recursive and interdependent process of environmental factors (e.g., family demographics or stereotypes) and individual-level factors of the subject (e.g., self-concept, identities, and personality) is built, which should eventually help explain an individual’s career decisions. Sense of belonging has not yet been systematically researched regarding the model, although its association to its main factors has been shown (Goodenow, 1993: value and success expectations in school subjects; Gillen-O’Neel and Fuligni, 2013: academic value).

In sum, belonging is an essential part of educational settings. As college belonging alone was found to be one of the main determinants for completing college (Yeager et al., 2016), belonging has an impact on students’ academic outcomes and can influence future career paths. Nevertheless, not every form of belonging is beneficial, such as feeling belonging to the group of females in a predominantly male environment such as physics. The consequences of belonging to a minority group, especially in environments in which achievements are relevant, have been researched and discussed in the literature on stereotype threat.

### Negative Consequences of Stereotype Threat for Women and Girls in Science

Women and girls in science are faced with negative stereotypes about them, and these negative stereotypes threaten both their belonging to the group and confirmation of the stereotype that goes along with their gender identity (see Schmader et al., 2015). Within the broader concept of social identity threat, stereotype threat theory stipulates that when a person identifies with a minority group that is negatively stereotyped by another group, the negative stereotypes can inhibit the person from performing at his or her best (e.g., Steele and Aronson, 1995; Spencer et al., 1999; Steele et al., 2002; Hall et al., 2015; Bedyńska et al., 2018). A variety of minority groups have been shown to be negatively affected by stereotypes pertaining to them (e.g., Aronson et al., 2002; Good et al., 2003; Hartley and Sutton, 2013; Froehlich et al., 2016); even men, when working in typically female environments, showed negative attitudes toward work and stronger intentions to opt out of their work field due to stereotypes (Kalokerinos et al., 2017). Females in STEM generally and in physics especially are among those negatively affected (e.g., Miller et al., 2015; Smyth and Nosek, 2015) due to the prevalence of harmful science gender stereotypes. Members of the stereotyped out-group, in this case girls and women in STEM, are often doubting their belonging to the in-groups. This is because of the characteristics attributed to the out-group (Cohen and Garcia, 2008). Typical stereotypes state that girls (out-group) have less talent for science subjects than males, which is why they might not belong to the science group (in-group). Consequently, girls not only show worse performance than their fellow male students (Shih et al., 1999; Flore and Wicherts, 2015) but also report feeling a lack of acceptance, more incompetence, and mental exhaustion (Hall et al., 2015), or even burnout (Hall et al., 2018). Whereas stereotyped groups were shown to exert more effort to prove the stereotypes about their social group wrong (e.g., Jamieson and Harkins, 2007), the effects were shown to be based not only on the social identity of and membership in the stereotyped group but also on the individual’s self. Therefore, using fictitious names and thereby separating performance from the identity of the participant enabled a reduction in stereotype threat effects on women (Zhang et al., 2012).

Intervening into the potentially vicious cycle of stereotype threat is important (Yeager et al., 2016), as forming a strong identity in an academics’ social system and confirming its values predict the persistence in a field (Estrada et al., 2011). Within this cycle, the connection of belonging and stereotypes becomes apparent again; by doubting their skills and abilities, students are sensitive to judging other’s behavior for cues that might be indicative of their doubt about one’s membership to the in-group (Aronson and Inzlicht, 2004), which leads to distancing oneself from the tasks and environment. This distancing can lead to feeling belonging uncertainty. Belonging uncertainty is the feeling of uncertainty of ones’ membership to the in-group (Walton and Cohen, 2007). Eventually, this uncertainty leads to negative consequences of stereotype threat, which range from lower performance (Steele and Aronson, 1995), impacting cognitive variables – such as more negative perception of one’s competence (Schmader and Johns, 2003) or even hindering girls and women from forming abilities in the stereotyped domain (Appel and Kronberger, 2012) – to affective variables – such as arousal or anxiety (Ben-Zeev et al., 2005). Interventions to interact in this cycle of negative consequences have been suggested and tested (see Schmader and Hall, 2014; Schmader et al., 2015).

Beyond this, stereotype threat was also shown to reduce girls’ interest in pursuing STEM in school or college, long-term interest, and career aspirations. Schmader et al. (2004) showed that women were more affected by stereotype threat when they personally endorsed stereotypes about women having less talent for and as a consequence had less intention of pursuing mathematics and less performance self-esteem and were not as confident in their abilities as women who did not endorse the stereotypes. Also, the threat affects girls and women regardless of whether the cues to stereotypes are implicit or explicit (e.g., Spencer et al., 1999; Marchand and Taasobshirazi, 2013). Stereotypes and belonging apparently interact throughout the entire education and work life; beginning in school, stereotype endorsement impacts task value and competence beliefs, thereby affecting career intentions (Plante et al., 2013), and negative stereotypes continuously influence careers in work life by negatively impacting organizational commitment in science careers (Block et al., 2018) and reducing older employees’ belonging (Rahn et al., 2020). Additionally, belonging uncertainty also interacts with the sense of belonging in a field. Uncertainty of belonging was found to depend on how participants assessed their own abilities in comparison to that of other students. Additionally, female students’ perceived exclusion, both on affective and academic levels, and self-efficacy in the field were further predictors of uncertainty of belonging in the field (Höhne and Zander, 2019). At the college level, it was shown that stereotype threat can hinder women in science from forming social connections to other students, even more so when having lower belonging (Martiny and Nikitin, 2019). Seeing that female STEM college students’ intentions to continue studying mathematics were predicted by sense of belonging and that stereotypical beliefs of the students’ environment about women’s abilities in mathematics led to less belonging for women but not for men (Good et al., 2012), belonging and stereotype threat appear to interact toward a disadvantage for women.

In sum, among other things, negative stereotypes about the abilities of women and girls in STEM cause a reduced sense of belonging (e.g., Good et al., 2012) and lower intentions to continue in the field (see Meyer et al., 2015). Thereby, the negative stereotypes about women but not men in physics affect women regardless of whether stereotypes are endorsed or not (Schmader et al., 2004; Good et al., 2012).

### Extracurricular Science Competitions as a Stereotypical Science Environment

In analyzing career aspirations in science and researching reasons for the gender gap, making a division between school and general interest seems reasonable, as it was found by Potvin and Hasni (2014b) that interest in science in school decreases throughout schooling, whereas interest in science in general increases. Students who have a strong interest in science and want to pursue that interest beyond what is offered as part of the school curriculum can participate in extracurricular activities, such as science fairs or science Olympiads. In Germany, the science Olympiads are popular and cover a wide range of science fields, such as biology, chemistry, or physics; in the International and European Junior Science Olympiad, Biology, Chemistry, and Physics Olympiads, and a national contest for environmental projects (BundesUmweltWettbewerb), 9,065 German students participated in 2019. Even though students voluntarily participate in these competitions, the underrepresentation of females in some of these disciplines – which is widely known from college and careers – is also on display in the Olympiads, especially so in the Physics Olympiad. Over four consecutive rounds, an ever decreasing number of participants compete for membership in the national team representing the country in the annual international competition. Although there is already a pronounced gender gap in the first round in the Physics Olympiad (28% of the first round participants in 2018 in Germany were female), the number of girls decreases disproportionally higher than the number of male participants in successive rounds. In 2019, similar to most of the previous years, all five members of the German national team were male. Little research exists on stereotype or social identity threat for high-performing or highly interested students in science. Ganley et al. (2013) researched stereotype threat effects for high-performing middle school students and, although they found no evidence of a stereotype threat effect in mathematics, they nevertheless showed lower performance by girls. Conversely, Bedyńska et al. (2018) showed that highly gender-identified girls were suffering more from stereotype threat, along with showing lower achievements and higher learned helplessness. Nevertheless, it was also shown that high-performing minorities that identify strongly with the domain are more likely to drop out of the domain (Osborne and Walker, 2006). Apparently, higher interest and identification may make students more susceptible to negative stereotypes (see Stone et al., 2012; Spencer et al., 2016) and might cause distancing from characteristics of the stereotyped identity (Pronin et al., 2004). Schmader et al. (2015) explain that whereas a consequence of stereotype threat might be more investment into the situation to prove the stereotypes wrong, feeling threat to belonging might lead to less investment or opting out of the domain.

The Physics Olympiad shows the typical male dominance and thereby one of the assumed stereotype threat consequences. Although they are interested in physics, girls do not continue along that path in physics, do not perform up to their full potential, and end up dropping out of the contest. Nevertheless, the phenomenon of stereotype threat has not yet been researched in this context. Also, the predominantly male environment might signal to women that they do not belong. This lack in sense of belonging, in return, could adversely influence their success and value beliefs according to the expectancy-value theory. Sense of belonging itself as a link between stereotype threat and the expectancy-value theory has not been investigated in the context of science competitions.

### The Current Study

**Figure 1:**
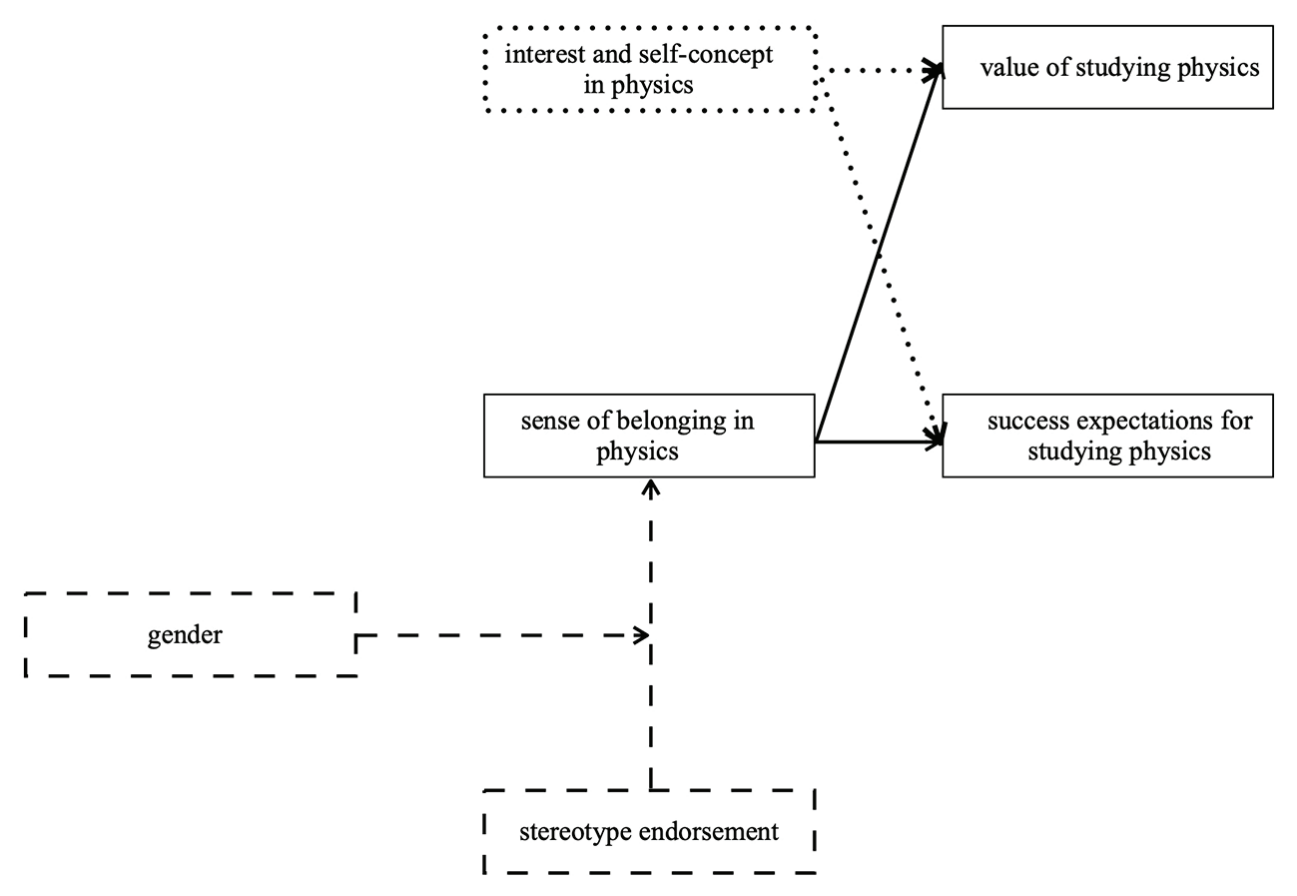
Researched model. Dashed lines show the possible predictors, that are hypothesized to change sense of belonging, and continuous lines show the hypothesized connection to the expectancy-value model, whereas dotted lines show the expected covariates.

Even though out-of-school programs seem less affected by the prevalent problem of declining science interests of students (Potvin and Hasni, 2014b), the Physics Olympiad nonetheless seems to represent a prototypical physics environment in which fewer women choose to engage and then leave the competition earlier and in larger numbers than their male counterparts. The present study addresses this problem by investigating sense of belonging as an important factor contributing to adolescents’ career choices in physics (e.g., Eccles et al., 1983; Eccles, 2009) and in the context of prevalent science stereotypes and stereotype threat for women (see Figure 1 for a research model). Based on previous literature, the following hypotheses were formed to investigate the research question, if girls participating in extracurricular science competitions are negatively impacted by stereotypes not just in the competition but also in their career aspirations:

1. *Hypothesis* 1:We hypothesize that endorsing stereotypes about girls and women in physics negatively affects sense of belonging of the female Physics Olympiad participants, whereas the male participants’ sense of belonging should not be affected.

The lens of stereotype threat theory (e.g., Spencer et al., 2016) was applied regarding this first hypothesis. We investigated the extent to which females experience less belonging than their male counterparts (e.g., Good et al., 2012) and whether they are adversely affected in their belonging by endorsing the stereotype, whereas there should be no similar adverse effect for the male participants (e.g., Schmader et al., 2004). The choice of participating in an extracurricular science environment suggests that participating students are interested in the field and want to engage beyond taking the subject in school. This leads to a highly selective group of participants, which is especially interesting regarding the female participants. The female students did not just decide to remain in the field but actively chose to continue in physics, thereby at least resisting previous cues that might have suggested girls should not be in science. Whether these females are nevertheless affected by stereotypes or suffer from stereotype threat has, to our knowledge, not yet been investigated. Previously, this has only been researched for school and college students and without connection to sense of belonging.

1. *Hypothesis* 2: We hypothesize that sense of belonging is impacting success expectations in and the value of choosing a career in physics for participants of the Physics Olympiad.

Based on previous evidence on success expectations and career aspirations in STEM fields (e.g., Kang et al., 2019; Selimbegović et al., 2019), regarding Hypothesis 2, we apply the lens of the expectancy-value theory (e.g., Wigfield and Eccles, 2000). The expectancy-value theory includes belonging as a predictor of career choices but has so far not systematically addressed the extent to which sense of belonging, above and beyond domain-specific self-concept and interest in physics, predicts expectancy- and value-related outcomes. We investigate the extent to which sense of belonging in the group of talented students within the Physics Olympiad predicts participants’ success expectations and value of choosing physics as a university subject or pursuing a career in physics.

#### Materials and Methods

The present study addressed first-round participants of the German Physics Olympiad, which is a national contest for non-university students under 20 years old. By means of achievement-based selection on written and experimental tests, the number of participants is reduced over four rounds, until only five participants are selected for the national International Physics Olympiad team. Participants in the contest have to solve tasks on their own (either at home or in school) in rounds 1 and 2. In these rounds, they do not interact directly with other competitors. In rounds 3 and 4, students meet and compete in interaction with other candidates.

Stereotypes were not explicitly triggered to elicit stereotype threat effects. Instead, it was assessed whether students endorsed common stereotypes about women’s and girls’ talent for physics and science. The regular physics setting, which interested students encounter when pursuing physics, was thus not changed or adapted. It was thereby assessed if students were subject to stereotype threat in a regular physics setting.

### Participants

The current study was part of a larger project (Effects of science competitions for students, WinnerS) which evaluates factors influencing participants’ success in the science Olympiads in Germany. For the current study, only the data of the first-round are included, which means that participants were participating alone and not interacting with other participants. All 931 first-round contestants of the German Physics Olympiad were informed and invited to participate in the study *via* e-mail, letters, and posters. Participation was voluntary and anonymous; it did not have any positive or negative effect on the participation or success in the Physics Olympiad. As an incentive, participation in the project was connected to a lottery that gave out several prizes (e.g., iPads and gift certificates).

A total of 282 first-round contestants responded to the invitation (30.29% response rate) and constituted the sample for the current study. Of these, 84 participants indicated female gender (age: *M* = 16.25, *SD* = 1.04) and 174 indicated male gender (age: *M* = 16.22, *SD* = 1.15); 24 individuals did not indicate their gender. Participants did not differ from the overall sample regarding gender [*t* (1,189) = −1.60, *p* = 0.109] or class level [*t* (474.09) = 0.80, *p* = 0.427], but participants were significantly younger than the overall sample [*t* (423.71) = 4.58, *p* < 0.001; *M*_Physics Olympiad_ = 16.59, *M*_Study_ = 16.23] and performed significantly better [*t* (481.08) = −3.45, *p* < 0.001; *M*_Physics Olympiad_ = 24.83, *M*_Study_ = 27.07]. The significant difference in age was not regarded as problematic, as students still not significantly differed in the other characteristics. Especially the nonsignificant results in class level, suggesting the same amount of physics education, and gender, suggesting that the gender ratio was maintained, lead to this conclusion. Therefore, it can be assumed that participants in the study are reasonably similar to the overall sample of participants in the Physics Olympiad.

Informed consent was obtained from the parents after which students filled in the questionnaire online.

### Measures

#### Sense of Belonging

Sense of belonging was measured with a shortened 15-item version of Math Sense of Belonging Scale of Good et al. (2012) that was adapted to physics belonging. The scale (each item rated from 1 “strongly disagree” to 4 “strongly agree”) measures the extent to which an individual feels belonging to the physics field. The scale consists of five subscales measuring different aspects of belonging with three items each. The subscale *membership* focuses on the extent to which an individual feels part of the wider physics community or physics group (e.g., “When I am in a physics setting, I consider myself a member of the physics world.”); *negative affect* focuses on the feelings one has when participating in the environment (e.g., “When I am in a physics setting, I feel tense.”; reversely coded); *trust* focuses on trusting the setting not to be biased (e.g., “When I am in a physics setting, I trust the testing materials to be unbiased.”); *desire to fade* focuses on the wish of being active in the environment (e.g., “When I am in a physics setting, I wish I was invisible.”; reversely coded); and finally *acceptance* focuses on the feeling of being seen as a member by other participants of the environment (e.g., “When I am in a physics setting, I feel accepted.”). The scale was used without further division into subscales for the analysis, similar to Good et al.’s (2012); internal consistency for the overall scale was high (Cronbach’s alpha = 0.84).

In order to gauge the structural validity of our adapted sense of belonging scale, a confirmatory factor analysis for sense of belonging was calculated with Mplus (version 8.2, Muthén and Muthén, 2018). The assumed model of sense of belonging was a second-order factor, loading on its five latent subscales trust, negative affect (recoded), acceptance, desire to fade (recoded), and membership, each indicated by its three manifest items (see Good et al., 2012). The results showed a fit of χ^2^ (*df* = 85, *N* = 261) = 188.24, *p* < 0.001, CFI = 0.94, TLI = 0.92, RMSEA = 0.07 (90% CI = 0.06, 0.08), and SRMR = 0.08. According to Hu and Bentler (1999) these values represented at least an acceptable or optimal model fit. Taken together with the high internal consistency of the overall scale, combining all items into one value for sense of belonging for each individual was permissible.

#### Career Aspirations: Expectancy-Value-Related Outcomes

Value and success expectations for a career in physics (i.e., taking up physics as a university subject or choosing a job in physics) were measured with four items each from an adapted and shortened scale from Lykkegaard and Ulriksen (2016) and Eccles and Wigfield (1995). All items could be rated from 1 “strongly disagree” to 4 “strongly agree.” The value measures the personal importance one attributes to studying physics at university or choosing a job in physics (e.g., “When I study physics or choose a job in physics, it will mean a lot to me to be successful.”). Success expectations are the beliefs about succeeding in physics at university when choosing to study it or choosing a job in physics and being successful at it (e.g., “When I study physics or choose a job in physics, I expect that I will show good performance.”). Cronbach’s alpha for value was 0.67 and for success expectations 0.75.

#### Stereotype Endorsement

At the end of the questionnaire, stereotype endorsement was measured by four items adapted from Fennema and Sherman (1976) to science (e.g., “Men are naturally better in science.”). Items were rated from 1 “strongly disagree” to 4 “strongly agree.” Cronbach’s alpha was 0.74.

#### Covariates

Self-concept in physics as the competence one perceives as having in physics was measured by six items (rated from 1 “strongly disagree” to 4 “strongly agree”) from the German Program for International Student Assessment (PISA) questionnaire (OECD, 2009; e.g., “When I am having physics lessons, I easily understand new concepts.”). Cronbach’s alpha was 0.88.

Interest in physics was measured on a four-item subscale from Daniels (2008) (e.g., “I would not like to give up physics because I enjoy physics.”). Items were rated from 1 “strongly disagree” to 4 “strongly agree.” Cronbach’s alpha was 0.84.

#### Analyses

Investigation of the two hypotheses – first, the extent to which females had a lower sense of belonging and are also adversely influenced by gender science stereotypes and, second, the extent to which sense of belonging predicted expectancy- and value-related outcomes – was done *via* linear regression analyses performed with SPSS (version 23.0, IBM Corp, 2015).

Regarding the first hypothesis, dummy coding for gender was applied (0 = male, i.e., the reference group, and 1 = female). Specifically, we first regressed sense of belonging on the dummy of gender:

$$Belonging_{j}=\beta_{0}+\beta_{1}\;•\;Gender_{j}+r_{j}$$

Here, the intercept *β*_0_ denotes sense of belonging in the reference group (i.e., for males), whereas the slope of gender *β*_1_ indicates the extent to which females differ in their sense of belonging from the reference group. The second step included stereotype endorsement as a further predictor:

$$\begin{array}{l} Belonging_{j}=\beta_{0}+\beta_{1}\;•\;Gender_{j}+\\ \;\;\;\;\;\;\;\;\;\;\;\;\;\beta_{2}\;•\;StereotypeEndorsement_{j}+r_{j} \end{array}$$

Whereby, the slope *β*_2_ indicates the overall impact of stereotype endorsement on belonging for both males and females combined.

Finally, whether females are adversely influenced by gender science stereotypes was investigated by additionally including an interaction term of gender and stereotype endorsement (stereotype endorsement was *z*-standardized before multiplication):

$$\begin{array}{l} Belonging_{j}=\beta_{0}+\beta_{1\;}•\;Gender_{j}+\beta_{2}\;•\;StereotypeEndorsement_{j}\\ \;\;\;\;\;\;\;+\beta_{3}\;•\;\left(Gender_{j}\times StereotypeEndorsement_{j}\right)+r_{j} \end{array}$$

Hereby, the slope of stereotype endorsement, *β*_2_, indicates the relation to belonging for the whole group, whereas the slope of the interaction term, *β*_3_, indicates the extent to which females differ in their relation between stereotype endorsement and sense of belonging compared to the males.

## Results

### Descriptive Statistics

Mean values (Table 1) in sense of belonging, domain-specific self-concept, and interest were greater than the scale mean (i.e., 2.5 on a scale from 1 to 4). Stereotype endorsement was rather low, although not significantly different for male and female participants [*t* (182.73) = 0.46, *p* = 0.650, Cohen’s *d* = 0.07]. Female participants reported significantly lower sense of belonging [*t* (258) = 3.14, *p* = 0.002, Cohen’s *d* = 0.41], success expectations [*t* (170.54) = 4.58, *p* < 0.001, Cohen’s *d* = 0.57], and self-concept [*t* (258) = 2.30, *p* = 0.022, Cohen’s *d* = 0.31] than male participants. Male and female participants did not differ in value [*t* (141.36) = 1.18, *p* = 0.242, Cohen’s *d* = 0.16] or interest [*t* (156.42) = 1.82, *p* = 0.071, Cohen’s *d* = 0.24].

**Table 1:** Descriptive statistics of the analyzed scales, which were included in the study.

	*M*	*SD*	*M_male_*	*SD_male_*	*M_female_*	*SD_female_*	*t*-statistic	*p*	Cohen’s *d*
Sense of belonging	3.30	0.39	3.36	0.35	3.20	0.45	3.14	0.002	0.41
Success expectations	3.00	0.54	3.10	0.52	2.79	0.50	4.58	<0.001	0.57
Value	3.27	0.51	3.30	0.47	3.22	0.57	1.18	0.242	0.16
Self-concept	3.50	0.51	3.55	0.47	3.39	0.59	2.30	0.022	0.31
Interest	3.41	0.59	3.44	0.57	3.30	0.61	1.82	0.071	0.24
Stereotype endorsement	1.49	0.56	1.51	0.58	1.47	0.52	0.46	0.650	0.07

The means and SDs are presented for the overall sample, as well as separated by gender. The results of *t*-tests, which compared male and female participants’ data, are presented as well as Cohen’s *d*.

Correlations of the scales can be found in Table 2 and, additionally, separated for male and female participants in Table 3. In the overall sample, sense of belonging and stereotype endorsement were negatively correlated with a small correlation (*r* = −0.13, *p* = 0.031). Splitting the group by gender shows that females’ sense of belonging was significantly correlated with stereotype endorsement (*r* = −0.39, *p* < 0.001), whereas males’ was not (*r* = −0.01, *p* = 0.907).

**Table 2:** Correlations of the analyzed scales for the overall sample.

	1	2	3	4	5	6
1 Sense of belonging	1					
2 Success expectations	0.50^*^	1				
3 Value	0.42^*^	0.45^*^	1			
4 Self-concept	0.50^*^	0.59^*^	0.34^*^	1		
5 Interest	0.42^*^	0.40^*^	0.56^*^	0.45^*^	1	
6 Stereotype endorsement	−0.13	0.05	−0.02	−0.09	−0.12	1

* *p* < 0.001.

**Table 3:** Correlations of the analyzed scales separated by gender.

	1	2	3	4	5	6
Sense of belonging	1	0.49^*^	0.38^*^ [Table-fn tfn2]	0.51^*^ [Table-fn tfn2]	0.33^*^ [Table-fn tfn2]	−0.39^*^ [Table-fn tfn2]
Success expectations	0.48^*^ [Table-fn tfn2]	1	0.52^*^ [Table-fn tfn2]	0.58	0.37^*^ [Table-fn tfn2]	−0.16
Value	0.44^*^ [Table-fn tfn2]	0.41^*^ [Table-fn tfn2]	1	0.52^*^ [Table-fn tfn2]	0.52^*^ [Table-fn tfn2]	−0.14
Self-concept	0.47^*^ [Table-fn tfn2]	0.60^*^ [Table-fn tfn2]	0.22^*^ [Table-fn tfn2]	1	0.59^*^ [Table-fn tfn2]	−0.33
Interest	0.47^*^ [Table-fn tfn2]	0.39^*^ [Table-fn tfn2]	0.61^*^ [Table-fn tfn2]	0.35^*^ [Table-fn tfn2]	1	−0.19
Stereotype endorsement	−0.01	0.14	0.05	0.04	−0.08	1

* *p* < 0.001. Correlations of male participants’ assessments are presented under the diagonal and correlations of female participants’ assessments are above the diagonal.

### Gender Differences in Sense of Belonging and Gender-Specific Effects of Stereotype Endorsement

**Figure 2:**
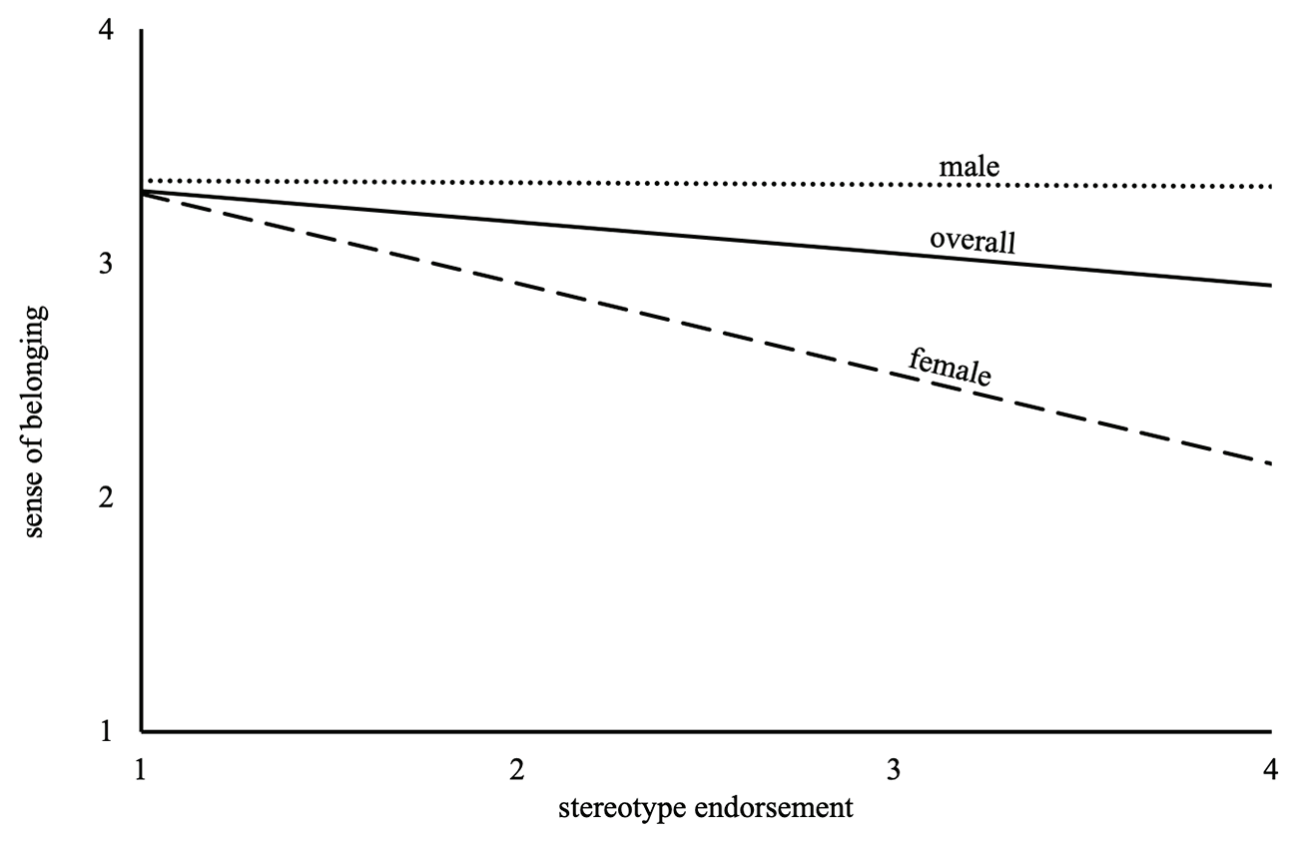
Graphic of the connection of sense of belonging and stereotype endorsement for female participants, male participants, and the overall sample.

A regression with dummy-coded gender (0 as male, 1 as female) was calculated (Table 4) to analyze data regarding Hypothesis 1, whether there were differences in females’ as compared to males’ sense of belonging. In the first step (Model 1), the slope for the gender dummy was statistically significant, indicating that female participants showed significantly lower belonging than male participants (*β* = −0.19, *p* = 0.002). In the second step of the dummy regression (Model 2), stereotype endorsement was added as a predictor of sense of belonging. The results evidenced an overall negative effect of stereotype endorsement on sense of belonging in physics (*β* = −0.14, *p* = 0.023). The interaction term of gender and stereotype endorsement was introduced to the model in the third and last step of the dummy regression (Model 3). The model showed that this overall relation between stereotype endorsement and sense of belonging was due to the females in the sample, who had a significantly stronger relation (*β* = −0.25, *p* < 0.001) compared to no relation for the males (Figure 2). The results thus go along with the hypothesis that female Physics Olympiad participants as opposed to male participants are negatively affected due to endorsing stereotypes about women and girls in science. The effect indicates that gender moderates the connection of stereotype endorsement and sense of belonging.

**Table 4:** Dummy regression analyses for stereotype endorsement on sense of belonging.

	Sense of belonging					
	Model 1		Model 2		Model 3	
Predictor	*β*	*SE*	*β*	*SE*	*β*	*SE*
Gender	−0.19	0.05	−0.20	0.05	−0.20^*^ [Table-fn tfn3]	0.05
Stereotype endorsement			−0.14	0.04	−0.01	0.05
Stereotype endorsement × gender					−0.25^*^ [Table-fn tfn3]	0.05
*R* ^2^	0.04		0.06		0.10	

* *p* < 0.001. Gender is coded male = 0 and female = 1.

### Sense of Belonging Predicting Success Expectations and Value for a Career in Physics

Linear regressions separately for success expectations and value for choosing physics at university or a job in physics were calculated to assess Hypothesis 2, whether sense of belonging predicted students’ career expectations. Sense of belonging was significantly predicting both variables (*β* = 0.50, *p* < 0.001 for expectancy; *β* = 0.42, *p* < 0.001 for value; Table 5). Even when controlling for possible confounding effects of interest and self-concept, which are known predictors for expectancy and value according to theory, sense of belonging was still a significant predictor for both success expectations (*β* = 0.25, *p* < 0.001) and value (*β* = 0.21, *p* < 0.001). Specifically, and in addition to sense of belonging (Model 1: *R* ^2^ = 0.25), in Model 2, self-concept in physics (*β* = 0.43, *p* < 0.001) but not interest (*β* = 0.10, *p* = 0.068) significantly predicted success expectations (*R* ^2^ = 0.42). The reverse was shown for value: additionally to belonging (Model 3: *R* ^2^ = 0.18), in Model 4, interest (β = 0.48, *p* < 0.001) but not self-concept (*β* = 0.03, *p* = 0.587) significantly predicted value of choosing to study physics or take a job in physics (*R* ^2^ = 0.38). These results are according to the hypothesis that sense of belonging predicts two important factors of the expectancy-value theory.

**Table 5:** Linear and multiple regression analyses for the expectancy-value model including models including covariates.

	Success expectations				Value			
	Model 1		Model 2		Model 3		Model 4	
Predictor	*β*	*SE*	*β*	*SE*	*β*	*SE*	*β*	*SE*
Sense of belonging	0.50^*^ [Table-fn tfn4]	0.07	0.25^*^ [Table-fn tfn4]	0.08	0.42^*^ [Table-fn tfn4]	0.07	0.21^*^ [Table-fn tfn4]	0.08
Self-concept			0.43^*^ [Table-fn tfn4]	0.06			0.03	0.06
Interest			0.10	0.05			0.48^*^ [Table-fn tfn4]	0.05
*R* ^2^	0.25		0.42		0.18		0.38	

* *p* < 0.001.

We added two further dummy regressions to analyze if gender also moderated between stereotype endorsement and interest or self-concept (Table 6), thus possibly having a further negative impact on career expectations for females in addition to its impact on sense of belonging. Following the three steps that were used to analyze the moderator previously, the dummy regressions were calculated. In the first step, gender significantly predicted self-concept (Model 1: *β* = −0.14, *p* = 0.022), showing that females had a lower self-concept in physics than their male counterparts. In the second step (Model 2), stereotype endorsement was included as a further predictor. Stereotype endorsement did not significantly predict self-concept (*β* = −0.09, *p* = 0.154), thus indicating that stereotype endorsement did not have an overall negative effect on self-concept. The interaction term of gender and stereotype endorsement was introduced to the dummy regression in the last step (model 3). It was significantly predicting self-concept (β = −0.23, *p* = 0.001). This shows that gender moderates between stereotype endorsement and self-concept. On the other hand, the moderation between stereotype endorsement and interest did not turn out to be moderated by gender (Model 3: *β* = −0.07, *p* = 0.344).

**Table 6:** Dummy regression analyses for stereotype endorsement on self-concept and interest.

	Self-concept						Interest					
	Model 1		Model 2		Model 3		Model 1		Model 2		Model 3	
Predictor	*β*	*SE*	*β*	*SE*	*β*	*SE*	*β*	*SE*	*β*	*SE*	*β*	*SE*
Gender	−0.14	0.07	−0.14	0.07	−0.15	0.07	−0.12	0.08	−0.12	0.08	−0.12	0.08
Stereotype endorsement			−0.09	0.06	0.04	0.07			−0.12	0.06	−0.08	0.08
Stereotype endorsement × gender					−0.23	0.07					−0.07	0.08
*R* ^2^	0.02		0.03		0.07		0.01		0.03		0.03	

Gender is coded male = 0 and female = 1.

### Structural Equation Model

**Figure 3:**
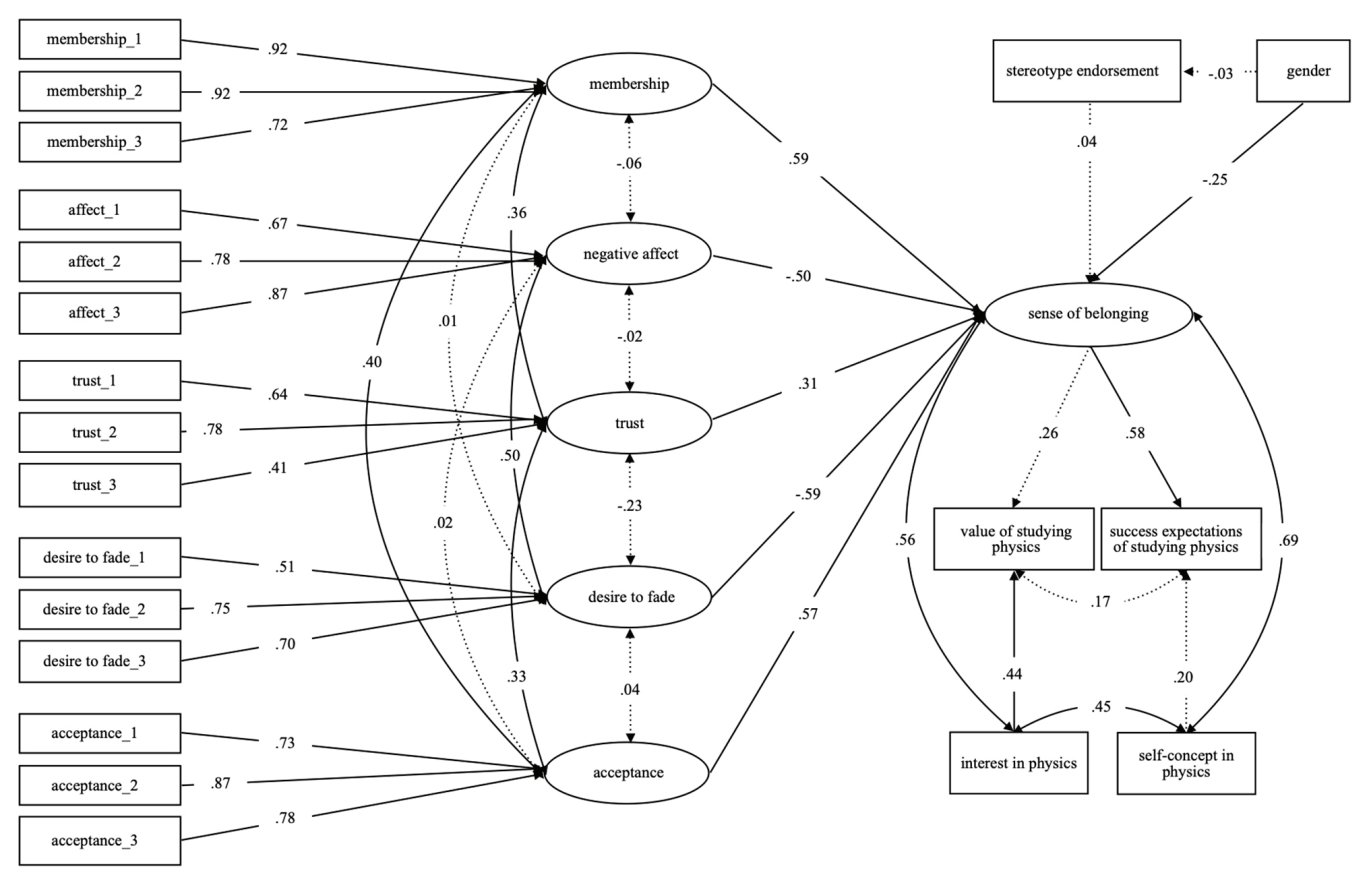
Structural equation model of the research model adapted to fit the previous results. Continuous lines show *p* < 0.001.

Lastly, the previous results were used to adapt the hypothesized research model in Figure 1 before testing the fit of the overall model with a structural equation model. The connection of success expectations for a career in physics with interest in physics and the connection of value of a career in physics with self-concept in physics were excluded from the model. The adapted structural equation model can be found in Figure 3. The model was tested using Mplus (version 8.2; Muthén and Muthén, 2018). The results show a model fit of χ^2^ (*df* = 169, *N* = 260) = 320.14, *p* < 0.001, CFI = 0.93, TLI = 0.91, RMSEA = 0.06 (90% CI = 0.05, 0.07), and SRMR = 0.07. According to Hu and Bentler (1999) this is a good fit. The model for the whole sample shows that sense of belonging is significantly predicted by gender (*β* = −0.17). Also, sense of belonging significantly predicts the value of choosing a career in physics (*β* = 0.26) additionally to interest (*β* = 0.44), as well as success expectations for choosing a career in physics (*β* = 0.58) additionally to success expectations (*β* = 0.20). The suggested model, which combines the impact of negative stereotypes and the expectancy-value theory, thus appears to fit within the Physics Olympiad context.

## Discussion

Research and research-based interventions need to explore how to retain the numbers of young women once they have decided on a physics career path. In the present study, the Physics Olympiad competition served as a prototypical physics environment; females not only are participating in lower numbers but also drop out of the competition in disproportionally higher numbers than the male participants. This paper addressed this problem by introducing sense of belonging as a previously rarely considered variable and situating it within an expectancy-value framework: female participants were found to have lower sense of belonging than male participants, indicating that the differences in belonging are due to the male-dominated environment signaling lower belonging by activating negative gender science stereotypes. However, above and beyond domain-specific self-concept and interest, sense of belonging predicted expectations and value of studying physics at university, two factors that, according to the expectancy-value theory, are predicting career choices. The adapted research model was shown to fit well, suggesting that belonging should be considered as an important factor within science career choices with regard to gender and the consequences of stereotype threat.

### Sense of Belonging and Stereotype Threat

The benefits of sense of belonging might be stifled by the problematic stereotype threat effects. Although participating female students were a group of highly interested and talented students, they nevertheless had a lower sense of belonging to the physics community than their male counterparts. Further, although there was no significant gender difference in endorsing the gender science stereotypes, girls’ belonging was significantly reduced the more they endorsed the stereotypes, whereas male participants were unaffected by endorsing stereotypes. We interpret this gender differential effect of stereotype endorsement on sense of belonging as stereotype threat in the physics competition, of which only female participants are susceptible. Adding to previous research on the field already showing lower sense of belonging for women in physics (e.g., Good et al., 2012) and women experiencing stereotype threat in science environments (e.g., Nosek et al., 2002; Schmader, 2002), the present study added insight regarding highly interested students. Even voluntarily choosing to be in a physics environment and joining with high levels of interest do not “immunize” girls to the detrimental effects of pervasive science gender stereotypes.

### Sense of Belonging and Career Aspirations

According to the expectancy-value model, success expectations and value are decisive for achievement-related choices, in this case choosing to study physics at the university level or choosing a job in physics. A rarely investigated variable in this process, beyond domain-specific self-concept and interest, is sense of belonging that connects the social environment to the individual’s need to belong to a group. The present study’s findings underscore the importance of sense of belonging in the school-to-university transition phase; sense of belonging to physics significantly predicted success expectations for studying physics at university and value of doing so. Sense of belonging had a predictive value above and beyond the expected influence of self-concept and interest (e.g., Nagengast and Marsh, 2012; Kang et al., 2019). Thereby, it is interesting to note that the three variables – belonging, self-concept, and interest – seem to serve different functions in forming future expectations and value beliefs: while self-concept but not interest predicted success expectations, and conversely interest but not self-concept predicted value beliefs, sense of belonging predicted both outcomes to a similar extent (Table 5). One conclusion is therefore that beyond an individual’s domain-specific beliefs, his or her belonging to a social environment also plays a crucial role in the formation of career decisions. In showing this incremental predictive validity of sense of belonging among a sample of highly interested students participating in an out-of-school physics competition, the present study adds to previous findings connecting sense of belonging to career aspirations (Good et al., 2012). It thus situates sense of belonging as an important additional predictor of achievement-related choices within an expectancy-value framework.

Seeing these results with regard to stereotype threat, they appear to emphasize the problems for females in science even more. Although the girls participating in the Physics Olympiad are interested and see themselves as capable of competing in physics, they are negatively affected by stereotypes. The stereotypes affect them negatively not just in their belonging but also in their self-concept, thereby possibly lowering their career aspirations even more.

### Limitations

Participation in this study was voluntary. Although there are only few differences regarding demographics between the German Physics Olympiad participants who partook in this study and those who did not, we cannot extend this conclusion to other motivational variables. However, even if only highly interested and engaged students participated in the assessment, this would not bias the interpretation of the present study’s findings that higher interest does not immunize against stereotype threat. We regard our findings as evidence that even among the highly interested students, females face adversity in physics, complementing previous research on stereotype threat. Further research should nevertheless look more closely into sense of belonging and note differences between school and extracurricular competitions as well as the differences in characteristics of students and participants in science competitions.

Further, all variables were measured at one time as the present study employed a cross-sectional design. Although we were guided by theoretical assumptions of underlying mechanisms, we strictly speaking cannot draw causal inferences. In the future, more research should focus on longitudinal or experimental designs to corroborate present findings pertaining to causal relations.

Finally, individuals suffering from stereotype threat due to negative stereotypes are usually inferred indirectly in previous studies, but not assessed by self-report measures. In our study, we inferred the existence of the stereotype threat effect by explicitly measuring stereotype endorsement, as previous research has shown that higher stereotype endorsement led to higher stereotype threat susceptibility (see Schmader et al., 2004; Pennington et al., 2016). However, explicit measurement of stereotype endorsement may lead to a social desirability bias; in fact, the overall endorsement of the gender science stereotypes was rather low in the current sample. Nevertheless, with regard to previous research, research on differing results between explicit and implicit measures delivers varying results, and implicit measures might not lead to other results in stereotype endorsement (e.g., Kessels et al., 2006). Thus, we have no indication that our gender differential effects could be biased by the explicit stereotype measure. Nevertheless, we believe that the present findings could be strengthened by adding an implicit measure of stereotypes in future research.

### Implications and Conclusion

The present study findings provide further insight into the pernicious hold that gender science stereotypes have over women and girls in STEM; stereotype threat not only reduces interest in pursuing physics overall but also specifically reduces belonging of those girls with an interest beyond normal curricular physics education and who engage in physics activities in their extracurricular leisure time.

From our findings, we draw two related implications:

1. Based on the finding that even highly interested young women participating in an out-of-school physics competition were susceptible to stereotype threat, which consequently lowered their sense of belonging, implies that competitions such as the Physics Olympiad need to address this in two possible ways: first, by adapting their environment in such a way that the gender science stereotypes or gender itself is made less salient in the achievement situation. Previously, three possible ways for this have been shown: using role models with regard to stereotypes in the domain (e.g., Cheryan et al., 2011), reducing the predominance of males (e.g., Inzlicht and Ben-Zeev, 2000), or changing cues in the environment that promote stereotypes (e.g., Murphy et al., 2007). Second, by strengthening females to make them less susceptible to stereotype threat, for instance, by teaching them about the necessity and ubiquity of struggles even of famous scientists (e.g., Lin-Siegler et al., 2016) or by strengthening their mastery mindsets through teaching them about the malleability of the brain (e.g., Blackwell et al., 2007). Interventions adapting the environment or strengthening individuals have previously shown good effects on students’ performance (e.g., Good et al., 2003) and intention to continue in science (e.g., Good et al., 2012).
2. Based on the finding that sense of belonging predicted career-related success expectations and value beliefs above and beyond domain-specific self-concept and interest implies that the feeling of belonging to an environment, being a member of it, valued and accepted, is an overlooked social component to motivation and career-related choices. Future research should dedicate itself in further situating sense of belonging within the expectancy-value framework and determine which features of the environment or personal characteristics further an individual’s sense of belonging.

In conclusion, this paper supports the idea that the expectancy-value theory and sense of belonging should be connected into one framework to systematically study and understand students’ physics career decisions. Belonging is a significant predictor of success expectations and value of studying physics at university both for females and males. Still, no matter how much they engage in physics in their extracurricular time, girls seem susceptible to stereotype threat and report lower sense of belonging to physics when they endorse negative stereotypes about females’ physics talent and ability. Therefore, promoting students’ interest by offering the opportunity to participate in out-of-school competitions such as the Physics Olympiad does nothing for a more equitable gender participation pattern in physics fields without further inclusion of gender-sensitive measures. To tap into the full potential of talented and interested young women in physics, the competition design and environment must counteract the pernicious effects of gender science stereotypes.

## Data Availability Statement

The raw data supporting the conclusions of this article will be made available by the authors without undue reservation.

## Ethics Statement

Ethical review and approval were not required for the study on human participants in accordance with the local legislation and institutional requirements. Written informed consent to participate in this study was provided by the participants’ legal guardian/next of kin.

## Author Contributions

AL wrote the first draft. AL, MK, and UK contributed to the interpretation of statistical analyses and edited and revised the manuscript. All authors contributed to the article and approved the submitted version.

### Conflict of Interest

The authors declare that the research was conducted in the absence of any commercial or financial relationships that could be constructed as a potential conflict of interest.
